# Tumoración Cardíaca con Complicación Embólica durante la Gestación

**DOI:** 10.47487/apcyccv.v1i1.14

**Published:** 2020-03-30

**Authors:** Romel Zamudio, Hector Vandyck, Zoe Díaz

**Affiliations:** 1 Servicio de Cirugía Cardiovascular Adulto - Instituto Nacional Cardiovascular INCOR. Lima, Perú. Servicio de Cirugía Cardiovascular Adulto Instituto Nacional Cardiovascular INCOR Lima Perú

**Keywords:** mixoma, embarazo, myxoma, pregnancy

## Abstract

Presentamos el caso de una mujer joven en el primer trimestre de un embarazo viable, complicado con infarto miocardio ST no elevado y mixoma atrial izquierdo. A pesar del riesgo que representa una cirugía cardiaca en una gestante, se decidió la exéresis del tumor cardíaco debido al alto riesgo embólico del caso. La cirugía se realizó sin complicaciones y controles posteriores mostraron viabilidad fetal hasta la fecha del parto.

## Abstract (en)

We present the case of a young pregnant woman in her first trimester, that presented with non-elevated ST myocardial infarction and left atrial myxoma. Despite the risk of cardiac surgery in a pregnant woman, the removal of the cardiac tumor was decided due to a high embolic risk. The surgery was performed without complications and following controls demonstrated fetal viability until delivery.

La incidencia de patologías cardiovasculares durante el embarazo es baja (1- 4%).^1^ Dentro de estas, los tumores primarios cardíacos son aun más raros, con una incidencia de 0,02%. Un 75% de estos tumores son benignos^2^ y en su mayoría son mixomas (tumores derivados del tejido conjuntivo). 

Casos de mixoma cardíaco en embarazadas son escasos en la literatura. Se presentan con signos o síntomas inespecíficos relacionados a su localización anatómica, tamaño, movilidad y efecto sobre las estructuras que lo rodean, siendo su localización más habitual el atrio izquierdo.^2^

En general el tratamiento quirúrgico temprano del mixoma es necesario debido a las posibles complicaciones cardiovasculares y embólicas fatales, pero si se presenta en una gestante es difícil decidir una resolución quirúrgica debido a las complicaciones que podría tener la circulación extracorpórea (CEC) en el equilibrio del binomio madre-feto.^1^

Se presenta el caso de una gestante con mixoma cardíaco que debutó con síntomas de isquemia cardíaca, el cual determinó la urgente indicación de remoción quirúrgica a pesar de los riesgos que la CEC condiciona en la viabilidad del feto.

## Descripción del Caso

Una mujer de 27 años, gestante de 11 semanas fue transferida a nuestra institución con diagnóstico de tumor cardíaco auricular, a descartar mixoma. La paciente estaba internada en otro hospital con el diagnóstico de infarto miocardio ST no elevado de 10 días de evolución, cuyo cuadro inició con dolor precordial (8/10) irradiado a brazo izquierdo y mandíbula al mínimo esfuerzo, cambios electrocardiográficos de cara inferior y elevación de enzimas cardiacas. 

**Figura 1:**
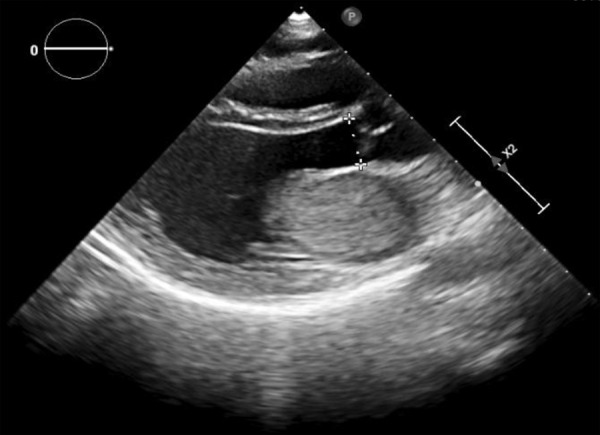
Ecocardiografía transtorácica en vista paraesternal eje largo: Presencia de tumoración en atrio izquierdo que se asienta sobre la válvula mitral.

**Figura 2:**
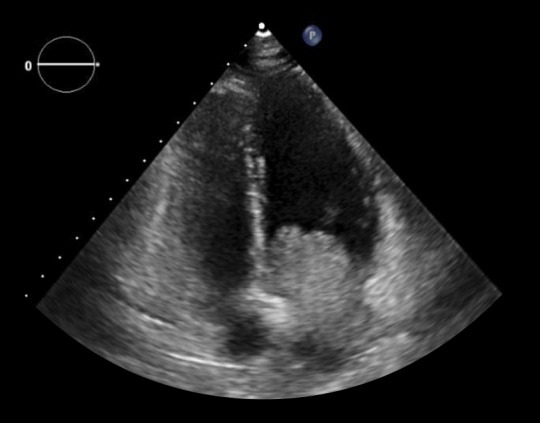
Ecocardiografía transtorácica en vista apical cuatro cámaras: Presencia de tumoración en atrio izquierdo que protruye hacia ventrículo izquierdo a través de válvula mitral.

En el electrocardiograma (EKG) de ingreso se encontró ritmo sinusal, onda T invertida y simétrica en derivaciones III y aVF. Se realizó ecografía transtorácica donde se encuentra una fracción de eyección del ventrículo izquierdo de 62%, una masa auricular en atrio izquierdo de 4 x 3 cm, móvil, que entra y sale al ventrículo Izquierdo con cada latido. (Figuras 1, 2 y 3) Los exámenes de laboratorio mostraron hemoglobina 14 g/dL, leucocitos 7.06 x 10^3^/μL, plaquetas 303 x 10^3^/μL, INR 1.94, creatinina 0.5 mg/dL, proteína C reactiva 2.9 mg/dL, creatina fosfoquinasa MB 14.7 U/L, troponina T 0,039 ng/dL. La evaluación de ginecobstetricia concluyó: gestación única activa de 11 semanas 2 días por fecha de última regla (FUR) y alto riesgo de compromiso de vitalidad fetal por posible cirugía cardiaca.

**Figura 3:**
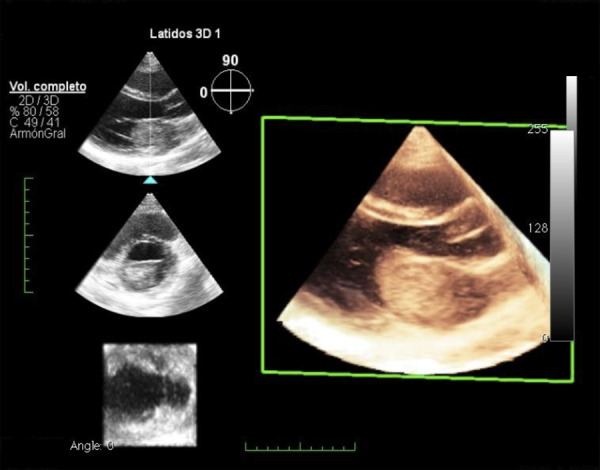
Ecocardiografía transtorácica: Volumen 3D que muestra la tumoración cardiaca en aurícula izquierda.

El equipo multidisciplinario, compuesto por cardiólogo, cirujano cardiovascular, anestesiólogo cardiovascular y ginecobstetra, decidió en junta médica la exéresis quirúrgica de la tumoración y posterior evaluación de la viabilidad fetal.

La cirugía se realizó mediante esternotomía media con canulación de aorta ascendente y bicava, parada con cardioplejía sanguínea con normotermia por vía anterógrada y atriotomía izquierda como abordaje. Se encontró una tumoración pediculada de 5 x 4 cm en atrio izquierdo, mixomatoide, friable, adherida a septum porción inferior. Tiempo de cirugía total: 2 horas y 45 minutos, tiempo de circulación extracorpórea: 40 minutos, tiempo de clampaje aórtico: 21 minutos.

La evolución post operatoria de la paciente fue muy favorable, con destete de la ventilación mecánica en el mismo día operatorio, y retiro de drenes mediastinales al día siguiente. Al quinto día, la paciente fue transferida estable a otra institución para su control ginecoobstétrico donde se corroboró la viabilidad del feto, sin signos de alarma. El informe anatomopatológico de la tumoración extraída concluyó como resultado: mixoma papilar. 

Por vía telefónica se contactó a la paciente quien refirió la evolución normal de su embarazo. Los controles ginecoobstétricos posteriores no evidenciaron ningún compromiso del feto, se le practicó cesárea dentro del tiempo de gestación convencional y el niño tiene un buen estado de salud. 

## Discusión

Los tumores cardíacos como los mixomas son raros en gestantes. El diagnóstico y tratamiento es un verdadero reto por su forma de presentación, complicaciones agregadas, y la disyuntiva del momento de cirugía cardiaca y la finalización del embarazo.^3^ En la mayoría de casos se diagnostica en el segundo trimestre pudiendo no tener síntomas inicialmente.^2^ Por otro lado, pueden presentar síntomas de obstrucción de la válvula mitral (estenosis mitral), insuficiencia cardiaca congestiva, embolismos sistémicos (siendo el más frecuente el cerebral), síntomas generales (fiebre, pérdida de peso y fatiga) y manifestaciones inmunológicas (mialgias, debilidad y artralgias). Algunos pueden ser confundidos y mal interpretados como síntomas asociados al embarazo,^4^ y por tal motivo es importante considerarlos, ya que se necesita un alto grado de sospecha clínica para decidir oportunamente su manejo.^2^

En términos generales, el tratamiento de elección es la remoción quirúrgica precoz cuando exista evidencia de complicaciones embólicas o de obstrucción severa de los tractos de entrada o salida ventriculares. Sin embargo, durante el embarazo, tratar quirúrgicamente un mixoma es complicado debido a los riesgos de la circulación extracorpórea, no solo por la respuesta inflamatoria sistémica con liberación de endotoxinas y activación de complemento, sino además por los cambios de temperatura, ausencia del flujo pulsátil fisiológico, variación de presiones durante el procedimiento, cambios del medio interno y del estado de coagulación, así como el uso de medicamentos normalmente necesarios en las cirugías cardíacas que bien podrían ser tolerados por la madre, mas no necesariamente por el feto.^5^^,^^6^

En ese sentido, algunos autores recomiendan realizar la cirugía luego del parto, ya que una cirugía durante el embarazo se asocia en 30% de casos a pérdida fetal, así como a riesgo de teratogénesis, alteraciones físicas y del desarrollo posnatal, contracciones uterinas y trabajo de parto. A pesar de ello, la tasa de supervivencia materna es cerca al 100%.^6^

La embolia cerebral en un mixoma intracardíaco izquierdo es mucho más frecuente que la embolia coronaria. A pesar de esto, existe la posibilidad de un infarto del miocardio. Mc Allister y Fenoglio hicieron revisión de tumores cardíacos en una serie grande y encontraron tres casos con cuadro principal de infarto del miocardio en 130 casos revisados.^7^

Las embolias coronarias pueden ser circunscritas a un territorio o ser masivas por embolias múltiples que también pueden estar relacionados a manipulación quirúrgica. Aunque se asume que los velos de la válvula aórtica protegerían de la embolización coronaria, se ha reportado casos de infartos en cara inferior correspondiente a la arteria coronaria derecha cuyo ostium se encuentra más anterior y superior que el ostium de la arteria coronaria izquierda.^8^

En nuestro caso, se decidió la exéresis del mixoma por el riesgo de embolia recurrente y por la evidencia de obstrucción del tracto de entrada del ventrículo izquierdo. Nuestra decisión al parecer fue la adecuada ya que la evolución postoperatoria fue satisfactoria tanto para la madre como para el feto.

## Conclusión

Los tumores cardíacos son raros durante la gestación y más aun la embolización coronaria. Su manejo es desafiante y requiere de un equipo multidisciplinario entre cardiólogos, cirujanos cardíacos y ginecoobstetras. El tratamiento quirúrgico se sugiere cuando hay riesgo de embolia sistémica u obstrucción significativa del tracto de entrada o salida ventriculares.

## Financiación:

Ninguna
